# Feasibility and Acceptability of an Active Video Game–Based Physical Activity Support Group (Pink Warrior) for Survivors of Breast Cancer: Randomized Controlled Pilot Trial

**DOI:** 10.2196/36889

**Published:** 2022-08-22

**Authors:** Maria C Swartz, Zakkoyya H Lewis, Rachel R Deer, Anna L Stahl, Michael D Swartz, Ursela Christopherson, Karen Basen-Engquist, Stephanie J Wells, H Colleen Silva, Elizabeth J Lyons

**Affiliations:** 1 Department of Pediatrics-Research The University of Texas MD Anderson Cancer Center Houston, TX United States; 2 Department of Kinesiology & Health Promotion California State Polytechnic University, Pomona Pomona, CA United States; 3 Department of Nutrition, Metabolism and Rehabilitation Sciences Sealy Center on Aging The University of Texas Medical Branch Galveston, TX United States; 4 Department of Biostatistics and Data Science The University of Texas Health Science Center at Houston, School of Public Health Houston, TX United States; 5 Department of Nutrition, Metabolism and Rehabilitation Sciences The University of Texas Medical Branch Galveston, TX United States; 6 Department of Behavioral Science The University of Texas MD Anderson Cancer Center Houston, TX United States; 7 Department of Surgery The University of Texas Medical Branch Houston, TX United States

**Keywords:** physical activity, survivors of cancer, pilot study, breast cancer, video games, group intervention, physical function, motivation, mobile phone

## Abstract

**Background:**

Survivors of breast cancer with functional limitations have a 40% higher mortality rate than those without. Despite the known benefits of physical activity (PA), <40% of survivors of breast cancer meet the recommendations for PA. The combination of active video games (AVGs) and group-based PA counseling may hold potential for motivating PA adoption and improving physical function. However, this method has not been widely studied in survivors of breast cancer.

**Objective:**

We aimed to determine the feasibility and acceptability of a group AVG-based multicomponent PA intervention and estimate its effect size and variability on PA and physical function in female survivors of breast cancer in a clinic setting.

**Methods:**

Female survivors of breast cancer (N=60) were recruited through the clinic and randomly assigned to the intervention group (12 weekly sessions) or the control group (existing support group). The intervention group received game-based pedometers and participated in weekly group AVG sessions, PA behavioral coaching, and survivorship navigation discussions. A participant manual with weekly reflection worksheets was provided to reinforce the coaching lessons and promote self-led PA. The control group received conventional pedometers and participated in an existing breast cancer support group. Feasibility was assessed by enrollment rate (≥50%), retention rate (≥80%), group attendance rate (75% attending ≥9 sessions [intervention group]), and the number of technological issues and adverse events. Acceptability was measured by participants’ attitudes (from strongly disagree=1 to strongly agree=5) toward the use of AVGs and the overall program. The outcomes included PA (accelerometers) and physical function (Short Physical Performance Battery and gait speed). Analysis of covariance was used to determine differences in PA and physical function between the groups. The Cohen *d* and its 95% CI determined the effect size and variability, respectively. All the analyses followed the intention-to-treat principle.

**Results:**

Participants were an average of 57.4 (SD 10.5) years old, 70% (42/60) White, and 58% (35/60) off treatment. The enrollment rate was 55.9% (66/118). Despite substantial long-term hurricane-related disruptions, we achieved an 80% (48/60) retention. The intervention group’s attendance rate was 78% (14/18), whereas the control group’s attendance rate was 53% (9/17). Of the 26 game-based pedometers, 3 (12%) were damaged or lost. No study-related adverse events occurred. Acceptability items were highly rated. Steps (*β*=1621.64; *P*=.01; *d*=0.72), Short Physical Performance Battery (*β*=.47; *P*=.01; *d*=0.25), and gait speed (*β*=.12; *P*=.004; *d*=0.48) had a significant intervention effect.

**Conclusions:**

The intervention was feasible and acceptable in this population despite the occurrence of a natural disaster. Pilot results indicate that group AVG sessions, PA coaching, and survivorship navigation produced moderate effects on PA and physical functioning. AVGs with PA counseling can potentially be used in existing breast cancer support groups to encourage PA and improve physical function.

**Trial Registration:**

ClinicalTrials.gov NCT02750241; https://clinicaltrials.gov/ct2/show/NCT02750241

## Introduction

### Background

With advances in the diagnosis and treatment of breast cancer, there are currently >3.8 million female survivors of breast cancer from diagnosis to end of life [1] living in the United States [2]. This means that more than 1 in 5 individuals with a history of cancer are female survivors of breast cancer [2]. Emerging evidence has shown that cancer and cancer treatment can exacerbate age-related deficits in physical function [3]. Without intervention, physical function limitations can lead to a cascade of functional decline, resulting in the loss of independence and early mortality [4]. In fact, a cohort study showed that survivors of breast cancer with functional limitations have a 40% higher mortality rate than those without functional limitations [5]. Therefore, intervening to prevent or reduce functional deficits could produce lasting benefits for the quality of life (QOL) of survivors of breast cancer [6].

Physical activity (PA) is a key approach to mitigating functional decline and improving QOL [7]. However, in the American Cancer Society (ACS) Study of Cancer Survivors-II survey, only 37.1% of survivors of breast cancer met the recommendations for PA of 150 minutes of moderate-intensity activity per week [8]. In another cohort study of 631 women, the percentage of survivors of breast cancer who met the PA guidelines decreased from 34% (before diagnosis) to 21.4% (10 years after enrollment) [9]. Furthermore, survivors of breast cancer were found to be similarly inactive or even more inactive than the general population or other populations of patients with chronic conditions [10]. Moreover, there is a growing concern that survivors of breast cancer are experiencing accelerated aging [3,11], which may also decrease their function. Thus, there is a critical need to develop PA interventions to help promote activity and prevent functional decline.

Although many successful behavior-based PA interventions have been effective in helping survivors increase their activity, these interventions are not without limitations [12]. First, a review of 51 behavior-based PA interventions found that as many as 62% were implemented in only 1 setting—individual or group-based—and only 17% used a group design [12]. However, survivors of breast cancer have indicated a need for interventions that offer a mix of individual and group-based settings [13]. Second, these interventions have not been widely integrated into clinical practice or community settings, so there is a need to test more scalable intervention models [10]. Third, the use of behavior change theories such as self-determination theory (SDT), a theory of motivation, coupled with technology-based tools (eg, active video games [AVGs]) to specifically target PA motivation has not been widely studied [14-17].

By meeting basic psychological needs (competence, autonomy, and relatedness), SDT posits that it will help promote autonomous motivation and increase PA [18]. Emerging research has shown that meeting basic psychological needs creates an autonomously supportive environment, which in turn increases the autonomous motivation to engage in PA in survivors of breast cancer [17,19,20]. Autonomous motivation, a motivation that arises from within the individual, includes identified regulation (ie, valuing PA and accepting the behavior as their own), integrated regulation (ie, being active is consistent with their sense of self), and intrinsic motivation (ie, motivation because of activity enjoyment) [18]. These types of motivation are needed for an individual to adopt and maintain PA behavior [20,21].

Among the few studies on group-based PA interventions is a recent meta-analysis that found that survivors who participated in group- and behavior-based PA interventions showed greater improvement in physical function than those who participated in an individual-based PA intervention [22]. Group- and behavior-based PA interventions also produced increases in PA participation and effort [23,24]. Moreover, group- and behavior-based activity interventions provide psychosocial benefits (eg, QOL and social support) that differ from those of individual-based interventions [25]. Our multicomponent PA intervention was designed to address this limitation by delivering the intervention in a group setting combined with a self-led component.

The wide implementation of an evidence-based PA intervention in clinics and communities could effectively address the need for PA during and after cancer treatment in survivors of breast cancer [26]. Given the numerous support groups available for survivors of breast cancer, support groups offer a potential setting for wide integration and dissemination. However, the combination of AVGs in groups, PA behavioral coaching, and breast cancer support has not been widely studied. Thus, there is a need to test this multicomponent design to accelerate the integration of a PA intervention into breast cancer support groups.

Among survivors of breast cancer, commonly cited reasons associated with the decline in activity level are fatigue, physical discomfort, and lack of belief in their ability to be active again (known as self-efficacy) [13,27,28]. Given that these challenges can affect how survivors of breast cancer respond to PA interventions, reframing PA as pleasurable may promote more motivation for the adoption of PA and lead to more effective PA interventions. AVGs may be a potential *gateway* method to motivate PA adoption and improve physical function among survivors of breast cancer [29,30]. Using AVGs to promote PA has several advantages. First, AVGs are designed to promote physical movement and can be used to facilitate low-cost and flexible PA interventions [31,32]. AVGs have the potential to be a cost-effective way to deliver a PA program in the community setting as they do not require someone to learn exercise moves before leading the sessions and they provide a variety of movement contents for the facilitator to select from [33]. Second, AVGs can encourage light to moderate PA and lead to better enjoyment of those activities when used as a tool to promote PA [34,35]. Third, PA duration increases in the intervention context despite discomfort, and the intention to participate in non-AVG PA also increases [34,35]. Finally, many AVGs include evidence-based behavior change techniques, such as those used in behavioral interventions, that are effective in promoting PA [36]. Examples of behavior change techniques used by AVGs include goal setting, feedback on PA progress, encouraging social comparison and interaction, and providing rewards [36]. In addition, the behavior change techniques used by AVGs can be used to target the basic psychological needs for autonomy, competence, and relatedness in SDT [18].

Previous studies that used AVGs in survivors of breast cancer and other survivors of cancer demonstrated that AVG interventions improved physical function (eg, muscle strength, range of motion, and QOL) [37-39]. However, the primary focus of previous studies was on the reduction of functional impairment based on the International Classification of Functioning, Disability, and Health model and QOL [37-40]. Owing to this focus on function, previous interventions did not include PA behavioral coaching, which is critical for promoting the adoption of PA behavior [12]. Furthermore, previous studies have yielded limited data on how AVGs affect the amount of PA [37-39]. Taking together the evidence in the literature, we have designed a unique method of delivering a multicomponent PA intervention to promote PA and physical function in survivors of breast cancer.

### Objectives

The primary aims of this pilot study were to (1) determine the feasibility and acceptability of a clinic-based multicomponent PA intervention (*Pink Warrior*) with a combination of AVG group play, group PA behavioral coaching, and breast cancer support (ie, survivorship navigation) and (2) determine the effect size and variability of the intervention on PA and physical function in female survivors of breast cancer. To our knowledge, the combination of AVG group play, PA behavioral coaching, and breast cancer support has yet to be tested in female survivors of breast cancer.

### Theoretical Framework

We adapted the *Pink Warrior* PA behavioral coaching materials based on the Active Living After Cancer (ALAC) program [41]. We also integrated the National Coalition for Cancer Survivorship (NCCS) Cancer Survival Toolbox and the Personal Health Manager kit of the ACS into the breast cancer support component [42]. Similar to the World Health Organization 2020 Guidelines on Physical Activity and Sedentary Behavior, the ALAC program focuses on adding PA to daily living through a group-based approach that teaches behavior change strategies and skill building. ALAC was tested in a randomized controlled trial [41] and expanded to clinical and community settings in another study [43]. Both studies found an improvement in the physical functioning of survivors of breast cancer after the intervention. Participants reported less pain and less daily activity limitation. Functional assessment indicated an increase in distance for the 6-minute walk test and in the amount of sit-to-stand activity completed in 30 seconds [41,43]. *Pink Warrior* adapted the ALAC program to include AVG technology to introduce various forms of PA and written materials that would allow minimally trained breast cancer support group facilitators (eg, social workers and graduate students) to implement the *Pink Warrior* intervention. Given previous research, we hypothesized that the multicomponent intervention would be feasible and acceptable for female survivors of breast cancer.

**Figure 1:**
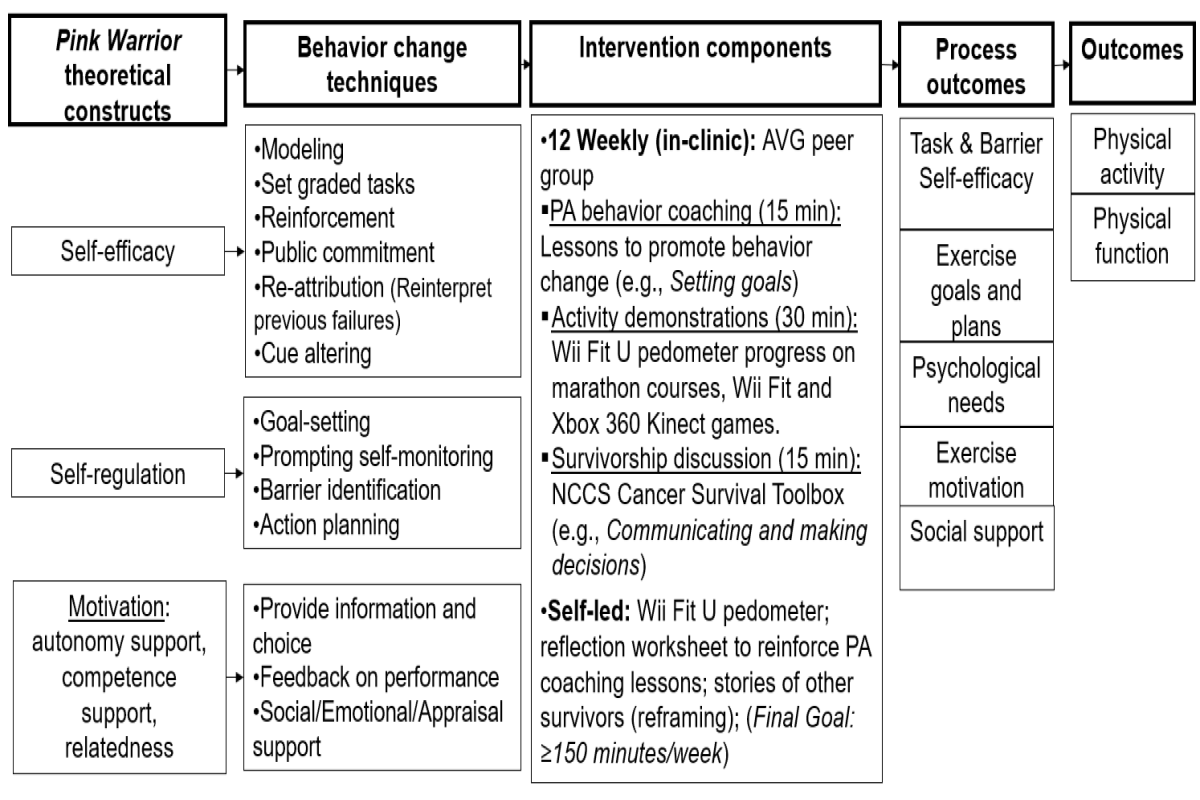
Pink Warrior logic model. AVG: active video game; NCCS: National Coalition for Cancer Survivorship; PA: physical activity.

Although the focus of this study is not the theoretical framework, methods, and components we used to develop the intervention, we have included the following summary to facilitate future replication of our *Pink Warrior* intervention [44]. Figure 1 shows the *Pink Warrior* intervention logic model, which summarizes the selected theoretical constructs, selected behavior change techniques, intervention components, process outcomes, and final outcomes of interest. The *Pink Warrior* intervention was based on the constructs of the social cognitive theory [27] and SDT [18]. Under social cognitive theory, we focused on the self-efficacy and self-regulation constructs. Self-efficacy and self-regulation are associated with the initiation of and increase in PA [45]. However, researchers have found that increasing autonomous motivation under SDT is important to promote PA over time [20]. On the basis of SDT, meeting the basic psychological needs for autonomy, competence, and relatedness will encourage autonomous motivation and lead to an increase in PA [46]. Thus, the *Pink Warrior* intervention was designed to increase participants’ autonomous motivation to engage in PA by targeting the self-efficacy, self-regulation, autonomy, competence, and relatedness constructs. The behavior change techniques we selected to target the theoretical constructs central to the *Pink Warrior* intervention included modeling, reinforcement, cue altering, goal setting, self-monitoring, action planning, barrier identification, and providing feedback on performance (Multimedia Appendix 1 [17,47]). Behavior change techniques are observable and replicable active ingredients used to target theoretical constructs and elicit behavior change [12,46]. We used the behavior change technique taxonomy developed by Michie et al [48] to align the behavior change techniques with the selected theoretical constructs. We chose these specific behavior change techniques because a systematic review demonstrated their effectiveness in targeting the theoretical constructs and increasing PA [44,49].

## Methods

### Participant Enrollment

**Figure 2:**
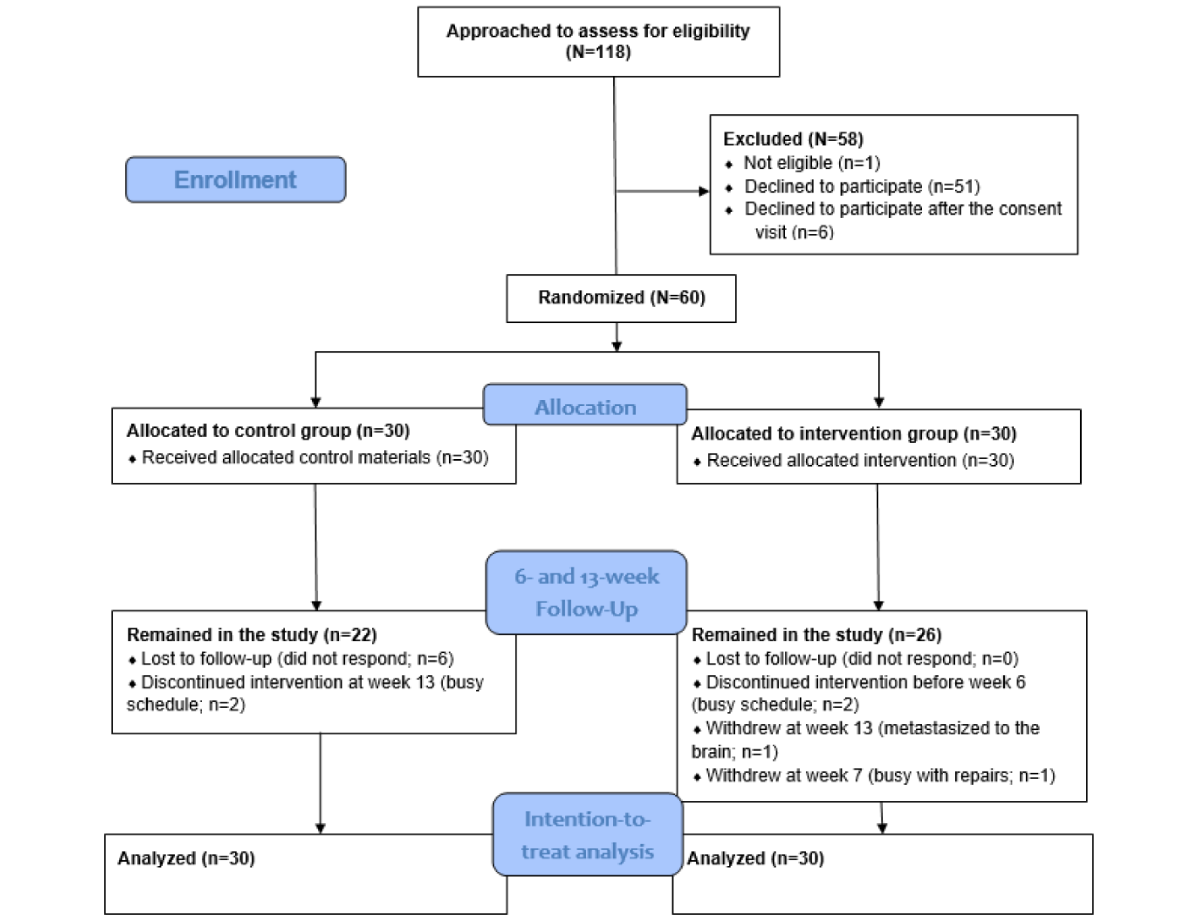
CONSORT (Consolidated Standards of Reporting Trials) pilot and feasibility flow diagram.

Our pilot study reporting was prepared in accordance with the CONSORT (Consolidated Standards of Reporting Trials) 2010 statement for randomized pilot and feasibility trials [50]. The CONSORT diagram of this study is shown in Figure 2. We conducted a phase 1b parallel pilot randomized controlled trial in which we used a 1:1 group allocation [51]. Participants (N=60) were recruited in 3 cohorts of 20 between July 2016 and July 2018 by mailing to registries through university announcements, flyers passed out within local cancer support groups, in-clinic flyers, and in-clinic recruitment. Cohort 1 was recruited over 6 months in 2016, and cohort 2 was recruited over 9 months in 2017 because of substantial long-term disruption to the lives of individuals in the recruitment area caused by widespread flooding from Hurricane Harvey. Cohort 3 was recruited over 6 months in 2018. The major inclusion criteria were age ≥18 years at diagnosis; a breast cancer diagnosis; ability to read, write, and understand English; approval from oncologists to participate; and ability to see a television screen from 2 to 4 feet. The major exclusion criteria included self-report of engaging in ≥150 minutes of planned moderate PA per week during the previous week, being currently involved in another PA intervention, or health issues that precluded safe participation. We purposely used less restrictive inclusion criteria to emulate the inclusiveness of a breast cancer support group. A standardized screening script was used by research coordinators and graduate students to determine study eligibility.

### Participant Randomization

Participants were randomly assigned to the PA intervention that combined AVG group play, PA behavioral coaching, and breast cancer support (intervention group) or to participate in the existing breast cancer support group at the study clinic with a pedometer (control group). We used the randomization procedure previously reported by Lyons et al [52]. Briefly, a project staff member (SJW) who was not involved in the assessment used a random number generator [53] to preassign numbers 1 to 20 (cohort 1), 21 to 40 (cohort 2), and 41 to 60 (cohort 3) to either the intervention or control group. The same staff member then sealed each piece of paper with the group allocation in a standard opaque envelope with carbon paper and foil. SJW then randomly shuffled a stack of 20 sealed envelopes per cohort and numbered them sequentially according to the study identification number. The carbon paper was used to provide an audit trail. The interventionist would sign and date each envelope that she opened and save the inner paper with the group allocation and carbon-copied signature and date in the participant file. The foil was used to prevent the interventionist from seeing the group assignment inside the envelope.

### Procedure

**Figure 3:**
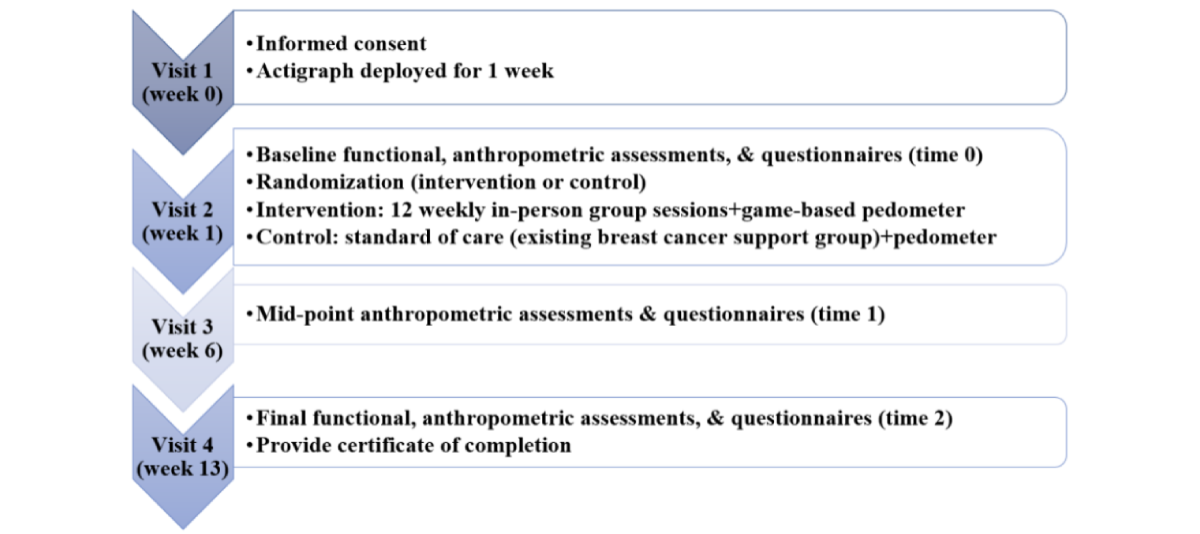
Study flow diagram.

All participants attended 4 scheduled informed consent and assessment visits. The study flow is summarized in Figure 3. The total study duration for each participant was 13 weeks, but the PA intervention duration was 12 weeks. Visit 1 was the informed consent visit. After informed consent was obtained, a research-grade activity monitor (*ActiGraph*) was provided for participants to wear for a week, and a packet of baseline questionnaires was provided for participants to complete before visit 2. Approximately 1 week later, participants returned for visit 2, where we conducted the full baseline functional assessment (time 0) and randomization and provided orientation for the study group into which the participants were randomized. Visit 3 was the midpoint assessment (time 1), which consisted of the completion of the questionnaires and PA assessment. The full final assessment occurred at visit 4 (time 2). Participants were not blinded to their group assignment. We were not able to conduct a blinded assessment owing to limited staffing resources. We obtained permission from the participants at the time of recruitment to send reminders via phone, SMS text message, or email to schedule appointments and the day before an appointment as a reminder.

### Ethics Approval

The Institutional Review Board at the University of Texas Medical Branch approved all procedures (protocol number: 16-0040), and our study was registered at ClinicalTrials.gov before data collection (NCT02750241).

### Intervention Group

The participants assigned to the intervention group took part in 12 weekly in-person, multicomponent PA intervention sessions. Participants were given a participant manual that contained weekly PA behavioral skill-building topics, self-led reflection worksheets, and breast cancer support discussion topics. Each of the in-person weekly group sessions consisted of three components: (1) a *PA behavioral coaching* (ie, *cognitive behavioral skill building*) component to promote the increase and maintenance of PA behavior, (2) an *AVG-based* *activity demonstration and practice* component to provide guided practice and increase mastery of activity skills using AVGs, and (3) a *breast cancer support discussion* component to provide support and resources for survivors of breast cancer. The weekly structured group session lasted approximately 60 minutes. A trained facilitator (graduate students pursuing a master’s degree or a research coordinator) would summarize the weekly PA discussion topic aimed at providing behavior change skills during the PA behavioral coaching component, help set up the AVGs, and facilitate the breast cancer survivorship discussions.

Within the *PA behavioral coaching component*, the adapted *Pink Warrior* intervention content focused on helping survivors of breast cancer overcome barriers to becoming more active and increasing self-regulation skills. The behavior change strategies included receiving feedback on PA, gaining knowledge regarding the benefits of PA, evaluating value toward activity, self-monitoring, goal setting, and action planning. These behavior change strategies addressed activity barriers such as lack of self-efficacy related to PA, lack of time because of competing demands, and lack of motivation because of general reasons or fatigue. The participants were asked to complete the reflection worksheet and accomplish the activity goals during the week following the PA behavioral coaching session. The reflection worksheets corresponding to the weekly coaching lessons were given to the participants to reinforce and encourage behavior changes toward self-led PA outside the group sessions.

AVGs that involved motion-controlled movement were used for the *AVG-based* *activity demonstration and practice component* of the *Pink Warrior* intervention. The AVGs were delivered through either the Wii Fit U (Nintendo EAD) game console or the Xbox 360 Kinect (Microsoft) game console in the group sessions. Participants only played the games in the weekly group session and were not given a Wii Fit U or Xbox console to use at home. Table 1 provides a summary of the types of games chosen by the study team in collaboration with the occupational therapist and the lymphedema therapy specialist at the study clinic. The fitness-based activities involved functional-based exercises such as balance exercises as well as body weight exercises and cardiovascular endurance exercises. As seen in Table 1, the fitness-based activities were chosen to introduce different activities that mimicked those that the participants could find to take part in on the web or in person.

**Table 1:** Examples of the games.

	Mind-body activities	Fitness-based activities
Wii Fit U	Walking game and yoga	Just Dance, Zumba, and dance games (Wii Fit U minigames and Your Shape Fitness Evolved 2012)
Xbox 360 Kinect	Your Shape Fitness Evolved 2012 (Zen energy, yoga, African rhythms, and Bollywood dance)	Your Shape Fitness Evolved 2012 (kickboxing; boot camp; and upper-, mid-, and lower-body training), Zumba, and Just Dance

As an example of social play, participants were able to track individual and group PA achievements in a gamified way during the weekly PA coaching session by wearing the Wii Fit U fitness tracker. Under the Wii Fit U game system, each participant was able to create an anonymous Mii character and select a marathon course (eg, the London marathon) that they would like to complete. Weekly, the accumulated steps recorded by the Wii Fit U fitness tracker were converted into the distance traveled (miles), and the participants were able to see themselves advance on the marathon course once the trackers were paired with the Wii Fit U game console. At the completion line, each participant’s avatar could then choose a new outfit with the destination’s design (eg, the London tower for the London marathon). Having the ability to see each other’s weekly accomplishments allowed for further enhanced motivation through social comparison and relatedness [31]. Participants were encouraged to meet their weekly step goals (eg, increase by 10% weekly) by seeking out AVG-based PAs that they found enjoyable in the community (eg, in-person or web-based tai chi classes or web-based videos) or by walking. Participants were given links to web-based videos of people playing the games to be used during the week. Paper-based PA logs were also provided for participants to record their activities on a daily, weekly, and monthly basis.

For the *breast cancer support component*, resources from the NCCS Cancer Survival Toolbox and ACS Personal Health Manager kit were used to elicit survivorship discussions. This component was designed to provide resources and support to survivors of breast cancer. Notably, it is the standard of care at the study clinic to provide the ACS Personal Health Manager kit to all new patients with breast cancer. However, the clinical team did not provide a detailed discussion on the content of the Personal Health Manager kit. Therefore, we integrated the NCCS Toolbox and ACS Personal Health Manager kit into the breast cancer support discussions to provide survivors of breast cancer with the tools to find credible resources.

### Control Group

Participants assigned to the control group at visit 2 (Figure 3) received the standard of care provided by the study clinic plus a step count monitoring intervention. The standard of care at the clinic included a monthly breast cancer support group that used its own materials. As part of the standard of care at the clinic, patients were also given the ACS Personal Health Manager kit, which included educational handouts related to PA during treatment and range of motion exercises developed by an occupational therapist who was also the lymphedema therapy specialist at the clinic. Furthermore, participants assigned to the control group were introduced to the facilitator of the existing breast cancer support group at the study clinic after the initial assessment. Control group participants were encouraged to take part in the clinic-based breast cancer support group every month until visit 4. The control group participants did not receive the *Pink Warrior* intervention or NCCS information while they were active in the study. However, the intervention materials were offered to the control group participants at study visit 4.

The step count monitoring intervention provided to the control group participants included a regular pedometer (*Omron HJ-321*) to be worn for the duration of the study period (between visits 2 and 4; Figure 3) [54]. During visit 2, we also helped control group participants set an activity goal and provided paper-based PA logs for them to record their activities on a daily, weekly, and monthly basis. We chose this type of control group intervention because of evidence related to the health benefits of PA among patients with and survivors of cancer [55]. In addition, similar interventions have also produced a short- and long-term increase in steps [56].

### Outcomes

#### Feasibility

Feasibility was assessed using the enrollment rate, retention rate, intervention group attendance rate, number of technological issues and adverse events reported by the research participants, and type of games played during the intervention group sessions. On the basis of typical outcomes of feasibility studies [57], we defined feasibility as the successful enrollment of at least 50% of the eligible participants approached or screened by the research coordinator and graduate students. The retention rate was feasible if at least 80% of participants completed the final assessment (Figure 3) based on previous PA or exercise studies conducted on survivors of breast cancer [57]. The group attendance or adherence rate was determined from the weekly or monthly attendance log maintained by the group facilitators. Group attendance was considered to be feasible if >75% of participants attended at least nine sessions in the intervention group. The number of technical and adverse events reported by the participants was determined using the participant database maintained by the study team. Information concerning the feasibility of the types of games played was obtained using a facilitator log.

#### Acceptability

The acceptability of the group AVG-based PA intervention components was measured using items adapted from Vandelanotte et al [58,59] and Lyons et al [52]. Acceptability was measured by participants’ agreement (from strongly disagree=1 to strongly agree=5) regarding the use of AVGs and the overall program. Participant acceptability and satisfaction data were collected at time 1 and time 2. Participant satisfaction was determined based on a questionnaire with 5-point scale responses. Participants were asked to report their satisfaction with the support time and length, intervention materials and staff, activity demonstrations, and discussion topics. They were also asked to provide written feedback at time 2.

#### PA Changes

The PA metrics examined in our intervention included average daily steps, average minutes of light PA, and average minutes of moderate to vigorous PA (MVPA). PA metrics were objectively measured using ActiGraph, a validated research-grade 3-axis accelerometer. The wear time was 7 days at each assessment point. As continuous measurement was not feasible, a week-long sample was taken at baseline, week 6 (–1 week to +1 week), and week 12 (–1 week to +1 week). We followed the accelerometer data processing protocol published by Keadle et al [60] for this pilot study, in which PA estimates were considered for analysis if the monitor was worn for at least 10 hours per day on at least one day. The step counters—Wii Fit U fitness tracker and Omron HJ-321—were used to promote self-monitoring behavior only.

#### Physical Function

The Short Physical Performance Battery (SPPB) was used to objectively measure physical function. The battery consists of 6 components: repeated chair sit-to-stand activity, balance test, semitandem stand, tandem stand, side-by-side stand, and 3-meter walk [61]. The handgrip strength test was objectively measured using the Jamar Digital Hand Dynamometer. Grip strength was assessed to measure changes in physical strength [26].

#### Self-reported Measures

Other self-reported measures included demographics such as age, gender, race and ethnicity, education, type of cancer diagnosis, and the type of treatment the participant was receiving. Self-reported measures were collected using paper-based questionnaires. The feasibility indicators were based on an enrollment and assessment database maintained by the study research coordinator. All other assessments took place face-to-face.

Participants did not receive any monetary incentives. Rather, a water bottle, a tote bag, and a T-shirt were provided to both the intervention and control group participants as a *thank you* or token of appreciation for participating in the study.

### Statistical Analysis

Data were analyzed using the SAS software (version 9.4; SAS Institute). Differences at baseline were investigated using Student *t* tests (2-tailed) and chi-square tests. Within-group comparisons between week 14 and baseline were performed using paired *t* tests. Differences between groups were estimated using analysis of covariance controlling for baseline values of the dependent variable and any baseline-intervention interaction (model: week 14 = [week 14 – week 0] + group + [week 14 – week 0] × group). The groups were coded as 0 (control) and 1 (intervention). Responses were missing at random and, thus, missing data were imputed using regression models [62]. The models consisted of the intervention status variable, the opposite time point, and 6 variables without missing observations. These 6 were selected from the 12 highest-ranked associations based on the prediction sum of squares statistic for each variable to be imputed. Multimedia Appendix 2 shows the imputation regression models for each variable imputed. For each outcome, the Cohen *d* [63,64] and its 95% CI determined effect size and variability, respectively. All statistical analyses used a significance level of .05. The primary purpose of this study was to evaluate the feasibility of the intervention components and study procedures to inform a larger intervention trial. Therefore, this study was not powered to detect a statistically significant difference in the PA and physical function outcomes. The statistical tests were conducted to provide estimated effect sizes and inform power and sample size estimates for the development of a follow-up intervention trial.

## Results

### Characteristics

As shown in Table 2, participants (N=60) on average were aged 57.4 (SD 10.5) years with a BMI of 30.6. Most participants (35/60, 58%) were off active cancer treatment at baseline, and the average time since diagnosis was 24.1 (SD 35.8) months. Of the 60 participants, 22 (37%) reported symptoms related to chemotherapy-induced peripheral neuropathy, and 33 (55%) had below-average grip strength for their age and gender at baseline [65]. No study-related adverse events were reported. We did not see any significant differences between the intervention and nonintervention groups related to the demographic characteristic variables.

**Table 2:** Participant characteristics at time 0 (N=60).

Characteristic		Total	Intervention (n=30)	Control (n=30)	*P* value^a^	
**Race and ethnicity, n (%)**						.31
	0—Non-Hispanic White	42 (70)	21 (70)	21 (70)		
	1—African American	10 (17)	3 (10)	7 (23)		
	2—Hispanic	4 (7)	3 (10)	1 (3)		
	3—Other	4 (7)	3 (10)	1 (3)		
**Stage, n (%)**						.79
	0	7 (12)	2 (7)	5 (17)		
	I	28 (47)	14 (47)	14 (47)		
	II	14 (23)	8 (27)	6 (20)		
	III	9 (15)	5 (17)	4 (13)		
	IV	2 (3)	1 (3)	1 (3)		
**Treatment type, n (%)**						.80
	Surgery only	11 (18)	5 (17)	6 (20)		
	Surgery and chemotherapy	9 (15)	5 (17)	4 (13)		
	Surgery, chemotherapy, and radiation	24 (40)	11 (37)	13 (43)		
	Chemotherapy only	2 (3)	2 (7)	—^b^		
	Chemotherapy and radiation only	2 (3)	1 (3)	1 (3)		
	Surgery and radiation	12 (20)	6 (20)	6 (20)		
**Current treatment status, n (%)**						.43
	Off treatment	35 (58)	16 (53)	19 (63)		
	On treatment	25 (42)	14 (47)	11 (37)		
**Self-reported symptoms related to chemotherapy-induced peripheral neuropathy, n (%)**						.59
	Yes	22 (37)	12 (40)	10 (33)		
	No	38 (63)	18 (60)	20 (67)		
**Grip strength below age and gender norm, n (%)**						.80
	Yes	33 (55)	17 (57)	16 (53)		
	No	27 (45)	13 (43)	14 (47)		
Age (years), mean (SD; range 29-80 years)		57.38 (10.48)	56.10 (10.65)	58.67 (10.33)	.35	
Time since diagnosis (months), mean (SD)		24.10 (35.83)	25.53 (39.14)	22.67 (25.62)	.74	
BMI (kg/m^2^), mean (SD)		30.62 7.46)	29.44 (6.24)	31.79 (8.46)	.22	

^a^*P* values calculated using the chi-square test for equal proportions for categorical variables and the 2-sample *t* test for continuous variables.

^b^No participants from the control group were in this category.

### Feasibility and Acceptability

The enrollment rate was 55.9% (66/118 eligible participants provided consent). In the intervention group, 13% (4/30) of the participants dropped out compared with 27% (8/30) of the participants in the control group (Figure 2). The most common reason for dropping out of the study was hurricane-related issues (eg, busy with house repairs). Despite substantial and long-term hurricane-related challenges, we achieved 80% (48/60) retention. Without accounting for missing sessions because of hurricane closures, participants in the intervention group attended a mean of 8.92 (SD 1.72) of the 12 sessions. We removed intervention participants (8/26, 31%) who were affected by the hurricane (eg, unable to attend because homes were flooded for weeks or severely damaged) from the adherence calculation to account for missing sessions because of hurricane closures, which produced a mean of 9.5 (SD 1.34) sessions. The intervention attendance rate was 78% (14/18 of included participants who completed at least nine sessions). In the control group, without accounting for missing sessions because of hurricane closures, participants attended a mean of 1.36 (SD 1.33) of 3 sessions. We removed control participants (5/22, 23%) who were affected by hurricane closures, which produced a mean of 1.56 (SD 1.37) sessions.

**Figure 4:**
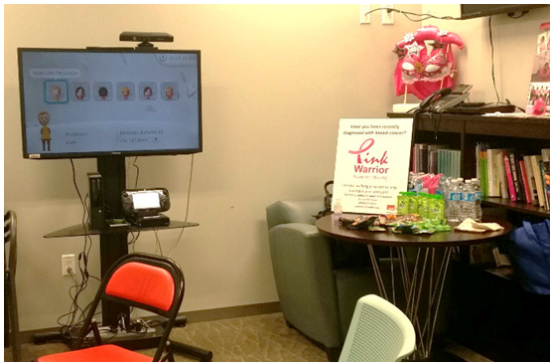
Room setup.

Of the 26 Wii Fit U game-based pedometers, 3 (12%) were damaged (eg, water damage) or lost. We were able to set up the game consoles in a small conference room (Figure 4). The location accommodated up to 4 participants and a facilitator at a time. On average, we formed 3 groups per cohort of 10 intervention participants because of room restrictions. Facilitator logs indicated that participants frequently selected mind-body activities (eg, tai chi and low-intensity dance games) during the first half of the intervention period (sessions 1-5) and progressed toward frequent selections of fitness-based activities (eg, Zumba and cardio boxing) during the second half of the intervention period (sessions 6-12). A total of 100% (60/60) of the intervention participants rated their acceptance of the *Pink Warrior* intervention at ≥4 on a scale of 1 to 5 (Table 3). Examples of postintervention feedback are included in Multimedia Appendix 3.

**Table 3:** Acceptability of the Pink Warrior intervention (time 2; N=26).

Item	Value, mean (SD)
Liked the Pink Warrior program	5.0 (0.2)
Appropriate activities	4.8 (0.4)
Program helped set reasonable goals	4.8 (0.5)
Contents were relevant	4.8 (0.4)
Program was worth my time and effort	5.0 (0.2)
Liked the contents presented (manual)	4.8 (0.5)
Liked the group setting	4.7 (0.6)
Liked the AVG^a^ portion	4.8 (0.5)
Liked the cancer survivorship topics	4.8 (0.4)
Like the program length	4.4 (0.9)
I would continue to participate	4.6 (0.7)

^a^AVG: active video game.

### PA and Function

The PA and objective physical function measurement results for the intervention and control groups are shown in Table 4. Intention-to-treat analyses in which missing data were imputed showed moderate effect sizes between the groups for PA outcomes such as the number of steps (*d*=0.72, 95% CI 0.20-1.24) and MVPA (*d*=0.72, 95% CI 0.19-1.24). For physical functioning outcomes, we found small between-group effect sizes, such as gait speed (*d*=0.48, 95% CI −0.03 to 0.99) and total SPPB score (*d*=0.25, 95% CI −0.26 to 0.75).

Analysis of covariance models controlling for baseline values of the dependent variable and any baseline-intervention interaction are shown in Table 5 and report the estimated *β* coefficients and corresponding *P* values from the Student *t* test. The results from our pilot intervention suggested that gait speed, total SPPB score, average daily steps, and MVPA had a significant intervention effect controlled for baseline and baseline-intervention interaction. Grip strength and light PA had nonsignificant intervention effects.

**Table 4:** Physiological effects of the intervention—mean of differences between baseline and final assessment for the intervention and control groups.

Variable	Intervention			Control			Effect size (between-group differences), Cohen *d* (95% CI)
	Mean of difference (SD)^a^	*P* value^b^	Mean of difference (SD)		*P* value		
Average grip strength	−0.094 (3.047)	.87	0.568 (1.826)		.10	0.26 (−0.25 to 0.77)	
Gait speed	0.109 (0.194)	.004	0.030 (0.131)		.23	0.48 (−0.03 to 0.99)	
Total SPPB^c^ score	0.653 (0.857)	<.001	0.421 (1.012)		.03	0.25 (−0.26 to 0.75)	
Steps	1556.200 (2614.8)	.003	−22.700 (1639.300)		.94	0.72 (0.20 to 1.24)	
Light PA^d^	13.322 (80.05)	.37	−10.687 (63.625)		.37	0.33 (−0.18 to 0.84)	
MVPA^e^	11.988 (18.994)	.002	0.999 (10.3444)		.60	0.72 (0.19 to 1.24)	

^a^Final assessment (time 2) – baseline assessment (time 0).

^b^*P* values indicate significant difference between final assessment and baseline.

^c^SPPB: Short Physical Performance Battery.

^d^PA: physical activity.

^e^MVPA: moderate to vigorous PA.

**Table 5:** Analysis of covariance results.

Variable	Week 0			Group or intervention status	
	*β* (95% CI; adjusted)^a^	*P* value	*β* (95% CI; adjusted)^a^		*P* value
Average grip strength	.806 (0.733 to 0.878)	<.001	−0.138 (−0.676 to 0.400)		.80
Gait speed	.838 (0.696 to 0.980)	<.001	.118 (0.079 to 0.157)		.004
Total SPPB^b^ score	.515 (0.428 to 0.601)	<.001	.470 (0.299 to 0.642)		.008
Steps	.915 (0.688 to 1.142)	.002	1621.637 (1063.480 to 2179.794)		.005
Light PA^c^	.899 (0.723 to 1.075)	<.001	21.014 (2.130 to 39.897)		.27
MVPA^d^	.414 (0.166 to 0.661)	.10	11.235 (7.672 to 14.799)		.003

^a^Adjusted for baseline treatment interaction.

^b^SPPB: Short Physical Performance Battery.

^c^PA: physical activity.

^d^MVPA: moderate to vigorous PA.

## Discussion

### Principal Findings

The aims of this study were to (1) describe the feasibility and acceptability of a clinic-based multicomponent PA intervention (*Pink Warrior*) with a combination of AVG group play, group PA behavioral coaching, and breast cancer support (ie, survivorship navigation) and (2) determine the effect size and variability of the intervention on PA and physical function in female survivors of breast cancer. Our results demonstrated that the group AVG-based PA intervention (*Pink Warrior*) was feasible and acceptable in a sample of middle-aged survivors of breast cancer who were on and off treatment. Evidence of feasibility was indicated by 55.9% (66/118) enrollment of eligible participants, 80% (48/60) retention at the end of the study, a 78% (14/18) adherence rate among intervention group participants, minimal technology issues, and no study-related adverse events. Evidence of acceptability was indicated by the mean acceptability scores exceeding 4 out of 5. The *Pink Warrior* intervention produced moderate effect sizes for PA metrics (ie, 0.72 for steps and 0.72 for minutes of MVPA) and a small effect size for objective physical function outcomes (ie, 0.48 for gait speed and 0.25 for SPPB score). We also found significant intervention effects on gait speed, total SPPB score, average daily steps, and MVPA. The effect sizes and significant intervention effects suggest that a larger-scale implementation of the intervention has the potential to produce a small to moderate effect and also reach minimal clinically important differences in PA and physical function metrics.

As previous AVG-based interventions in survivors of breast cancer did not specifically evaluate the feasibility and acceptability of the interventions, we compared our findings with other PA interventions conducted in survivors of breast cancer. Overall, our feasibility findings fall within the range of accepted values for PA interventions conducted in survivors of breast cancer [57] and also within the range of accepted values for AVG-based interventions conducted in individuals with cancer [66]. Our enrollment rate of 55.9% (66/118) was higher than the overall median enrollment rate of 45% across various PA interventions in survivors of breast cancer [57]. In addition, our overall 80% (48/60) retention was within the range of other AVG-based interventions in survivors of cancer (50%-100%) [66]. Similar to the findings of the systematic review conducted by Singh et al [57], we found a greater dropout rate in the control group than in the intervention group. Even though our adherence rate was lower than the overall median adherence rate (81%) reported by Singh et al [57], it is within the acceptable range for PA interventions in survivors of cancer on and off treatment (62%-96.6%) [66,67]. Most of our adherence issues resulted from the fact that 47% (14/30) of *Pink Warrior* intervention group participants were on treatment. Many of the missed sessions were because the participants were experiencing side effects from chemotherapy and were not able to travel to the in-person group AVG session. Most attrition issues during our study resulted from the post–Hurricane Harvey recovery burden on some participants. Mainland Galveston County, where the clinic-based sessions took place and where many participants lived, was among the hardest-hit areas during this extreme flooding event, resulting in substantial long-term disruptions.

In addition to its feasibility, the results of our AVG-based intervention indicated acceptability. The mean acceptability score of >4 is consistent with other exergame-based PA interventions [66]. Specifically, our intervention participants rated the content, group setting, and AVG portion of the intervention close to 5 out of 5. According to the facilitator log, intervention participants selected a variety of games from both the Wii Fit U and Xbox 360 Kinect game consoles. The high acceptability may be related to having PA variety, which enabled participants to try different activities that ranged from mind-body to fitness-based activities. Beyond the questionnaire feedback, acceptability was further demonstrated by how often participants joined either the weekly intervention group sessions or the usual clinic-based breast cancer support group. The intervention group participants attended 78% (14/18) of the scheduled AVG-based group sessions, whereas the control group participants attended 53% (9/17) of the scheduled clinic-based breast cancer support group sessions. Our results indicate that AVG-based activities along with PA coaching can potentially be added to the existing clinic-based support group to enhance engagement and participation among survivors of breast cancer who are on and off treatment.

Our PA outcomes indicate that the AVG-based intervention benefited the participants. The increase in average number of steps per day among the intervention participants was similar to that published by Sajid et al [68]. The increase of 1556.2 average daily steps among our intervention participants falls between the increases in average daily steps for the Wii intervention group in the study by Sajid et al [68] (+1223.8 steps per day) and their home-based walking and resistance intervention group (+19,414.4 steps per day) at the end of their 6-week intervention program. Similarly, the control group participants in both our study and the study by Sajid et al [68] experienced a decline in average daily steps (−22.7 and −383.4 steps per day, respectively) [68]. As there is limited published information on the influence of AVGs on PA levels, we further compared our findings with PA interventions that used wearables and smartphone apps [69]. Compared with the findings of Gal et al [69], our effect size for average daily steps was higher (*d*=0.72 vs *d*=0.51), as was our effect size for average minutes of MVPA (*d*=0.72 vs *d*=0.43). Beyond achieving a moderate effect size for average daily steps and average minutes of MVPA, the increase of >1000 steps per day estimated in 13 weeks also met the threshold for a minimal clinically important difference. A recent systematic review by Hall et al [70] found that an increase of 1000 steps per day among adults (mean age range 49.7-78.9 years) was associated with a decreased risk of all-cause mortality and cardiovascular disease–related morbidity or mortality. Hence, this finding is promising as it points to the effectiveness of a multicomponent PA intervention using AVGs for survivors of breast cancer during and after treatment.

The changes in the physical function outcomes among our PA intervention group are also promising. The intervention participants showed an increase in SPPB score (+0.653 in SPPB score) at the end of the intervention, whereas the Wii intervention group in the study by Sajid et al [68] did not show an increase in SPPB score. The difference between our intervention results and the finding of Sajid et al [68] suggests that the PA coaching that was integrated into our AVG-based PA intervention promoted engagement in activities that helped increase the total SPPB score. We were unable to locate other exergame interventions in survivors of cancer that specifically reported a change in gait speed [66]. However, the effect size found for gait speed as a result of our intervention, although small (0.11 m/s), showed a clinically important change. Evidence from the literature indicates that an increase of 0.11 m/s in gait speed is associated with a lower risk of morbidity and mortality [71]. The slight but not significant reduction in grip strength in our intervention group was a surprising finding. This finding may be related to several factors. First, we had more survivors of breast cancer who were on treatment in the intervention group than in the control group. A reduction in strength during cancer treatment has been established [72]. Second, the activities chosen mainly targeted lower-body functioning. Given that Sajid et al [68] showed an increase in grip strength with the use of resistance bands, resistance training could potentially be integrated into AVG-based PA interventions.

### Strengths and Limitations

Our phase 1b pilot randomized controlled trial had several strengths. First, it involved an innovative intervention design that paired group-based AVGs with PA behavioral coaching to promote PA behavior among survivors of breast cancer. We systematically designed the intervention by aligning the intervention components with behavior change methods and theoretical constructs. Previous studies that used AVGs in survivors of breast cancer and other survivors of cancer primarily focused on the reduction of functional impairment; thus, their focus was not on promoting the adoption of PA behavior [37-39]. Second, our use of objective measures of PA and physical function overcame some of the limitations (eg, overreporting and underreporting) that are associated with self-reported measures [73-75].

However, our study also had several limitations that are associated with the study design. First, this was a pilot study with a small sample size. Therefore, we were not fully powered to detect statistically significant differences in the participants’ outcomes or the long-term maintenance of PA behavior and physical function. Thus, our focus was on evaluating the effect size of the main outcome measures, which will provide the effect estimates needed to design a larger trial. Despite the small sample size, our AVG-based PA intervention produced moderate effect sizes and clinically important changes, which indicate that a larger-sample trial is worthwhile. Second, the pilot intervention was designed to test the feasibility and acceptability of the full intervention. The focus was on developing the most efficacious multicomponent program rather than on evaluating the impact of specific intervention components. Therefore, we were not able to determine the feasibility, acceptability, or effects of the individual portions of the intervention. Given that we found moderate effect sizes and clinically important changes in PA and physical functioning outcomes, a factorial-designed efficacy trial will be considered for a larger trial to determine the mechanisms of action of the intervention’s individual portions. Third, there was a difference in the number of group sessions offered to the control and intervention participants. This is because participants assigned to the control group received the standard of care provided by the study clinic plus a step count monitoring intervention. The highly advertised monthly breast cancer support group was a part of the study clinic’s standard of care. The differences in the number of sessions could potentially affect the differences in outcomes. Even so, the control group participants were provided with a step count monitoring intervention in addition to the standard of care to allow for activity tracking. A systematic review and meta-analysis found that similar interventions have also produced short- and long-term increases in steps. Therefore, the moderate effect sizes and clinically important changes found in our study would still be considered valid. Fourth, our *Pink Warrior* intervention involves more extensive facilitator training than the current support group format. Therefore, time for facilitator training may be an issue for future implementations. However, as the study has demonstrated feasibility, a subsequent study will be conducted to evaluate how to efficiently deliver facilitator training. Finally, this study was limited to the greater southeastern Texas community. Therefore, our pilot results may not be nationally generalizable. However, we will use our results to inform the design of a larger and more generalizable study.

### Conclusions

In summary, our results suggest that a clinic-based multicomponent PA intervention that combines AVG group play, group PA behavioral coaching, and breast cancer support (eg, survivorship navigation) is feasible and acceptable for middle-aged survivors of breast cancer on and off treatment. Given our results, the use of AVGs combined with manualized PA behavioral coaching can potentially be a scalable and promising strategy that can be integrated into existing breast cancer support groups to promote PA in survivors of breast cancer. Future studies are needed to understand how to efficiently integrate AVGs and PA behavioral coaching into existing breast cancer support groups. Through such integration, we will then be able to increase reach and deliver an evidence-based PA intervention to promote PA and enhance physical function. In addition, we need to better understand how and why AVGs help increase PA and physical function by comparing a group that includes AVGs with PA coaching and survivorship navigation with a group that only has PA coaching and survivorship navigation.

## Appendix

Multimedia Appendix 1 Summary of theoretical constructs, behavior change methods, and intervention components.

Multimedia Appendix 2 Models for imputation of missing data.

Multimedia Appendix 3 Examples of postintervention feedback.

Multimedia Appendix 4 CONSORT-eHEALTH checklist (V 1.6.1).

## Abbreviations

ACS: American Cancer Society
ALAC: Active Living After Cancer
AVG: active video game
CONSORT: Consolidated Standards of Reporting Trials
MVPA: moderate to vigorous physical activity
NCCS: National Coalition for Cancer Survivorship
PA: physical activity
QOL: quality of life
SDT: self-determination theory
SPPB: Short Physical Performance Battery
UTMB: University of Texas Medical Branch
