# Supplementary material for: Feasibility and Acceptability of an Active Video Game–Based Physical Activity Support Group (Pink Warrior) for Survivors of Breast Cancer: Randomized Controlled Pilot Trial
Source: JMIR Cancer. 2022 Aug 22;8(3):e36889. doi: 10.2196/36889 (PMC9446134; doi:10.2196/36889)
Supplement: Multimedia Appendix 1 [file cancer_v8i3e36889_app1.pdf]

Multimedia Appendix 1.

Table S1. Summary of theoretical constructs, behavior change methods, and intervention components.

**Theoretical construct[47]**	**Behavioral change method [17, 47]**	**Intervention components**	
		**AVG[a] support group**	**Existing support group**
**Task self-efficacy**	Modeling	Modeling of PA[b] behavior through the avatar in the AVG and other participants in the group	
	Set graded tasks	AVG (increase difficulty until target behavior is performed)	
	Reinforcement	Unlocking higher level AVG games	
	Public commitment	Group session—PA behavioral coaching goal setting discussions	
**Barrier self-efficacy**	Reattribution training (reinterpret previous failures)	Group session—PA behavioral coaching lesson	
	Cue altering	Group session—PA behavioral coaching lesson	
**Self-regulation**	Goal setting	Weekly log, reflection worksheets, group session—PA behavioral coaching lesson	
	Prompting self-monitoring	Wii Fit U meter, weekly log	Pedometer and weekly log
	Barrier identification	Group session—PA behavioral coaching lessons	
	Action planning	Weekly log, reflection worksheets, and group session—PA coaching lesson	
**Motivation**			
Autonomy support	Provide information using non-controlling	Non-directive group discussion, group game play (choose from a	ACS personal health manager kit (general

	informational language and provide choice	menu of games), ACS personal health manager kit	information)
Competence support	Offer constructive, clear, and relevant feedback	Tailored feedback through AVG: badges, unlocking the game, weekly progress chart based on the Wii fit meter, other participants' non-evaluative feedback/reaction to the progress made on a weekly basis, and process-focused feedback from the facilitator	
Relatedness	Social support	Group play, group discussion that prompts discussions regarding PA behavioral progress, and identification with other survivors	Identification with other survivors in the usual breast cancer support group
	Social support—emotional support (acknowledge and respect perspectives and feelings)	Group sessions related to PA behavioral coaching and discussion of survivorship issues, focus on empathy and acknowledgement of each participant's perspective	Group sessions on survivorship issues as presented in the usual breast cancer support group
	Social support—appraisal support	See the "friends'" progress in the group	

[a]AVG: active video game.
[b]PA: physical activity.
