# Supplementary material for: Feasibility and Acceptability of an Active Video Game–Based Physical Activity Support Group (Pink Warrior) for Survivors of Breast Cancer: Randomized Controlled Pilot Trial
Source: JMIR Cancer. 2022 Aug 22;8(3):e36889. doi: 10.2196/36889 (PMC9446134; doi:10.2196/36889)
Supplement: Multimedia Appendix 2 [file cancer_v8i3e36889_app2.pdf]

Multimedia Appendix 2.

Table S2. Models for imputation of missing data.

Variable to impute	Predictors
Average grip strength (baseline)	Total SPPB[a] score (baseline), PROMIS[b] physical function t-score (baseline), gait speed (baseline), PROMIS fatigue *t* score (baseline), FACT-B[c] total (baseline), autonomy (baseline), average grip strength (week 14), intervention group
Average grip strength (week 14)	PROMIS physical function *t* score (baseline), extrinsic regulation (baseline), integrated regulation (baseline), FACT-B total (baseline), gait speed (baseline), intrinsic regulation (baseline), average grip strength (baseline), intervention group
Gait speed (week 14)	Gait speed (baseline), total SPPB score (baseline), intrinsic regulation (baseline), PROMIS fatigue *t* score (baseline), PROMIS physical function *t* score (baseline), introjected regulation (baseline), intervention group
Total SPPB score (week 14)	Total SPPB score (baseline), gait speed (baseline), PROMIS physical function *t* score (baseline), extrinsic regulation (baseline), intrinsic regulation (baseline), introjected regulation (baseline), intervention group
Steps (baseline)	PROMIS physical function *t* score (baseline), FACT-B total (baseline), PROMIS fatigue *t* score (baseline), integrated regulation (baseline), intrinsic regulation (baseline), competency (baseline), steps (week 14), intervention group
Steps (week 14)	Integrated regulation (baseline), autonomy (baseline), PROMIS fatigue *t* score (baseline), gait speed (baseline), FACT-B total (baseline), competency (baseline), steps (baseline), intervention group
Light PA[d] (baseline)	FACT-B total (baseline), competency (baseline), Total SPPB score (baseline), PROMIS physical function *t* score (baseline), introjected regulation (baseline), autonomy (baseline), light PA (week 14), intervention group
Light PA (week 14)	Integrated regulation (baseline), autonomy (baseline), PROMIS fatigue *t* score (baseline), gait speed (baseline), FACT-B total (baseline), competency (baseline), light PA (baseline), intervention group
MVPA[e] (baseline)	Integrated regulation (baseline), identified regulation (baseline), intrinsic regulation (baseline), introjected regulation (baseline), FACT-B total (baseline), gait speed (baseline), MVPA (week 14), intervention group

MVPA (week 14)	Extrinsic regulation (baseline), Total SPPB score (baseline), introjected regulation (baseline), autonomy (baseline), PROMIS physical function *t* score (baseline), gait speed (baseline), MVPA (baseline), intervention group

[a]SPPB: short physical performance battery.
[b]PROMIS: Patient-Reported Outcomes Measurement Information System.
[c]FACT-B: Functional Assessment of Cancer Therapy-Breast.
[d]PA: physical activity.
[e]MVPA: moderate and vigorous physical activity.
