# Supplementary material for: Feasibility and Acceptability of an Active Video Game–Based Physical Activity Support Group (Pink Warrior) for Survivors of Breast Cancer: Randomized Controlled Pilot Trial
Source: JMIR Cancer. 2022 Aug 22;8(3):e36889. doi: 10.2196/36889 (PMC9446134; doi:10.2196/36889)
Supplement: Multimedia Appendix 3 [file cancer_v8i3e36889_app3.pdf]

Multimedia Appendix 3.

Table S3. Examples of postintervention feedback

**What I like the most about the Pink Warrior program**
"Cancer survivorship portion was good to get to know team and share your story without it being too much/lengthy." [Participant 4]
"The support was more important than I anticipated when I started. I am very thankful for my little group. It became a good emotional support!" [Participant 16]
"The program motivated me to become more active. I am now running with my family. I have increased from running 0.5 to 1 mile." [Participant 23]
"Without the weekly encouragement I would have never finished this trial. Now I am to keep up my daily workout." [Participant 24]
"It was a great group of ladies and it was nice to be able to learn about each other's trials and journey. They were all encouraging." [Participant 42]
"I enjoyed our conversations, making new friends, setting goals, and tracking miles we walked, it was fun." [Participant 52]
"The Wonder Woman costume kind [of] became my trademark, I wore it to Walk a mile in her shoes, a domestic violence awareness walk. I was [using] a walker at that time and my best friend—went with me...I owe the confidence I have gained to [the team]." [Participant 61]
**What I like the least about the Pink Warrior program**
"Needs to last longer" [Participant 17]
"Not long enough" [Participant 18]
"Time of the class was hard for me as a shift worker and I was usually around 10 minutes late, but the instructor was very nice about it." [Participant 28]
"The fast dance moves; only because I am not very coordinated" [Participant 42]
"Homework" [Participant 41]
"1 hour goes by too fast!" [Participant 57]
There wasn't anything that I didn't like [Participant 63]
